# Endothelial Cell Capture of Heparin-Binding Growth Factors under Flow

**DOI:** 10.1371/journal.pcbi.1000971

**Published:** 2010-10-28

**Authors:** Bing Zhao, Changjiang Zhang, Kimberly Forsten-Williams, Jun Zhang, Michael Fannon

**Affiliations:** 1 Department of Ophthalmology and Visual Sciences, University of Kentucky, Lexington, Kentucky, United States of America; 2 Department of Chemical Engineering, Virginia Polytechnic Institute and State University, Blacksburg, Virginia, United States of America; 3 Department of Computer Science, Laboratory for High Performance Computing and Computer Simulation, University of Kentucky, Lexington, Kentucky, United States of America; Vascular Health Research Centre, Ireland

## Abstract

Circulation is an important delivery method for both natural and synthetic molecules, but microenvironment interactions, regulated by endothelial cells and critical to the molecule's fate, are difficult to interpret using traditional approaches. In this work, we analyzed and predicted growth factor capture under flow using computer modeling and a three-dimensional experimental approach that includes pertinent circulation characteristics such as pulsatile flow, competing binding interactions, and limited bioavailability. An understanding of the controlling features of this process was desired. The experimental module consisted of a bioreactor with synthetic endothelial-lined hollow fibers under flow. The physical design of the system was incorporated into the model parameters. The heparin-binding growth factor fibroblast growth factor-2 (FGF-2) was used for both the experiments and simulations. Our computational model was composed of three parts: (1) media flow equations, (2) mass transport equations and (3) cell surface reaction equations. The model is based on the flow and reactions within a single hollow fiber and was scaled linearly by the total number of fibers for comparison with experimental results. Our model predicted, and experiments confirmed, that removal of heparan sulfate (HS) from the system would result in a dramatic loss of binding by heparin-binding proteins, but not by proteins that do not bind heparin. The model further predicted a significant loss of bound protein at flow rates only slightly higher than average capillary flow rates, corroborated experimentally, suggesting that the probability of capture in a single pass at high flow rates is extremely low. Several other key parameters were investigated with the coupling between receptors and proteoglycans shown to have a critical impact on successful capture. The combined system offers opportunities to examine circulation capture in a straightforward quantitative manner that should prove advantageous for biologicals or drug delivery investigations.

## Author Summary

In this work we have investigated the role of a family of cell surface molecules, proteoglycans, in blood vessel capture of proteins important to normal and diseased states under flow conditions. We developed a computer model to analyze and predict these events and, using an experimental system incorporating endothelial-lined hollow fibers as model blood vessels, tested our predictions. We found that both proteoglycans and flow exert significant influence over growth factor binding to the vessel wall. Removal of proteoglycans significantly reduced binding of these proteins; and flow rates slightly higher than that seen in capillaries had a similar effect, albeit in a different way. This knowledge will increase our understanding of interactions inside blood vessels and help to design more efficient pharmaceuticals. Also, our computer model has the potential to test the ability of existing and future drugs and biologics to successfully target blood vessels.

## Introduction

The bioavailability of molecules as they circulate through the bloodstream is a crucial factor in their signaling capability. Half-life in circulation can determine the effectiveness of a drug simply by regulating the opportunities a molecule has to interact with the vessel wall. Although *in vivo* measurements are routinely made by researchers to monitor serum levels of molecules and to determine half-lives, interactions in the microenvironment are not easily measured or observed. While some molecules may have a long circulation life, many may have only a single opportunity to interact with the blood vessel walls before being filtered through the liver or kidneys. In addition, even molecules with a long circulation life may still face impediments to direct interaction with the endothelium. This, for example, is the case with vascular endothelial growth factor (VEGF) when bound to bevacizumab, a monoclonal antibody to VEGF [1], [2]. Bevacizumab has been shown to increase the circulating concentration of VEGF in cancer patients when compared to patients not undergoing therapy because of the increased half-life of the growth factor-antibody complex; however the complex is unable to bind to VEGF receptors [3] making delivery of the VEGF questionable. In order to better understand the vessel microenvironment and to accurately monitor drug interactions in the context of that microenvironment, better tools are needed to provide meaningful measurements that can predict the fate of molecules in circulation.

Many important measurements have and continue to be made using *in vitro* mammalian tissue culture methods but there are obvious limitations to the traditional two-dimensional culture approach. In circulation, the influence of flow on whether a molecule remains in the fluid phase or binds to the vessel wall can be a dominant factor. This influence cannot be ascertained in static tissue culture studies. For example, the velocity of blood in the aorta is ∼400 mm/sec while at the capillary level it is less than 1 mm/sec [4]. This reduction in velocity allows the exchange processes at the capillary level to take place more efficiently [4] and it likely also affects the activity of molecules in circulation that rely on cell surface binding in order to fulfill their roles. While direct measurement of this binding process is difficult, our model makes use of a commercial bioreactor with endothelial-lined hollow tubes operating under pulsatile flow to mimic the vascular environment architecture and to directly measure the loss of molecules as they pass through these hollow fibers. We have used a single pass method to allow better assessment of the effect of flow in either retaining molecules in the circulation or permitting their interaction with vessels. Our approach also makes use of a bolus administration, since this is a typical way in which drugs would be delivered in a clinical setting.

The binding of fibroblast growth factor-2 (FGF-2) to its cell surface receptor (FGFR) and the role of heparan sulfate proteoglycans (HSPG) in regulating the process have been of research interest for many years because of their role in angiogenesis, the growth of new blood vessels from existing vessels. Knowledge of how these processes work could aid in the development of new therapeutics to control tumor growth and assist clinically in the treatment of chronic wounds. In order to understand the mechanism of FGF-2-mediated cell proliferation, a multitude of experimental studies have been undertaken [5] and, in the past two decades, several computational models of FGF-2 binding to its receptor FGFR and HSPG have been proposed [6]–[11]. Insight can be gained through experiment-coupled modeling that could not otherwise be readily obtained. Nugent and Edelman [11] were among the earliest researchers to develop a simple model that includes three species, FGF-2, FGFR and HSPG. They measured kinetic binding rate constants experimentally and used their model to analyze the data thereby providing a foundation for investigating the complexity of FGF-2 binding. A similar approach was used by Ibrahimi *et al* [9] to investigate stepwise assembly of a ternary FGF-2-FGFR-HSPG complex in conjunction with their surface plasmon resonance measurements. We introduced more complexity into the FGF-2 binding model with the inclusion of heparin binding [12], receptor dimerization [8], and formation of alternative HSPG-FGFR species [13]. Recent models have moved towards including intracellular signaling [14]. With the exception of work by Filion and Popel [7], [15], which included diffusive transport, previous simulation work has been based on a static tissue culture environment that may be quite different from the dynamic *in vivo* environment of blood vessels.

We introduced a computational model based on a flow environment in which the competitive binding of FGF-2, FGFR, and HSPG in a pulsatile flow environment was addressed to mimic blood vessel-like hollow fibers [16], [17]. In this paper we use an updated version of that model to explore how specific parameters such as flow rate impact FGF-2 capture and receptor binding, and compare our results with experimental studies. Insights with regard to the importance of surface coupling and ligand depletion zones within the fluid phase were found. The described simulation package provides a new and valuable way to investigate growth factor capture and can be easily extended to other biologically relevant molecules and drugs.

## Materials and Methods

### Preparation of Bovine Aortic Endothelial Cells (BAECs)

BAECs (passage 10), cryopreserved in liquid nitrogen, were cultured in Dulbecco's modified Eagle's medium (DMEM-low glucose, phenol red-free, Invitrogen Corporation, Grand Island, NY), supplemented with penicillin (100U/mL, Invitrogen Corporation, Grand Island, NY), streptomycin (100µg/mL, Invitrogen Corporation, Grand Island, NY), glutamine (2mM, Invitrogen Corporation, Grand Island, NY), and 5% newborn calf serum (Invitrogen Corporation, Grand Island, NY). When a sufficient number of cells were grown (passage 11∼13), they were transferred to the hollow fiber cartridge.

### Preparation and maintenance of endothelial cartridges

**Figure 1:**
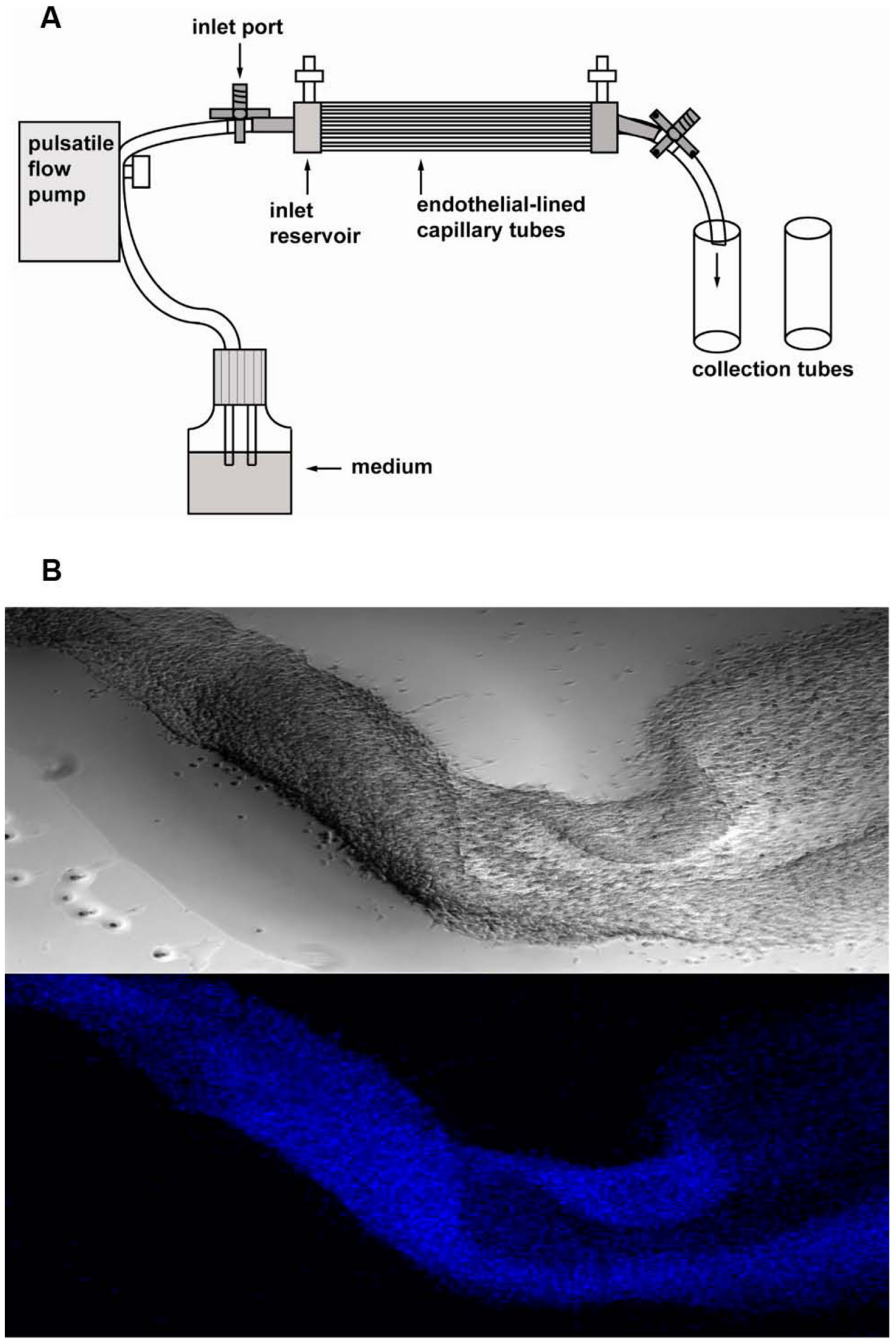
The bioreactor system. (A) A diagram of the experimental set-up, and (B) brightfield and DAPI stained images of endothelial cells from the unit showing the continuous vessel-type architecture.

The FiberCell polysulfone plus endothelial cartridges (C2025, FiberCell Systems Inc., Frederick, MD), also called hollow fiber bioreactors, contain 20 capillaries which are 12 cm long, 700 µm I.D., 300 µm wall, 0.1µm pore size, 53 cm^2^ lumen surface area (Figure 1A). They were activated with 70% ethanol (Fisher Scientific, Houston, TX), followed by multiple washes with sterile distilled water. The cartridges were then coated using 5 µg/mL fibronectin (Sigma Aldrich, St. Louis, MO) in phosphate buffered saline (PBS, Invitrogen Corporation, Grand Island, NY). BAECs (passage 11∼13) were inoculated into the cartridges (0.7–1×10^7^ cells/cartridge) 24 hours after the coating and placed in an incubator for 4 hours (rotated 180° after 2 hours) without flow in order to promote cell attachment. The BAEC culture cartridges were then linked to the FiberCell pump system (FiberCell Systems Inc., Frederick, MD) and media circulated through the system at ∼2.6 mL/minute (5.2 mm/sec). The flow system was maintained in the incubator (37°C, 5% CO_2_) at all times except during the experiment periods. Cell growth and viability was monitored by measurement of the cell glucose consumption from the medium once a day with OneTouch UltraSmart blood glucose monitoring system (Lifescan, Inc., Milpitas, CA).

### Growth factor flow studies

The flow system and cell-lined cartridges were removed from the incubator, gently washed once with warmed (37°C) PBS (60 mL), and then maintained in circulating 125 mL serum-free medium (DMEM-low glucose, phenol red-free, supplemented with 0.05% gelatin in PBS) in a sterile room-temperature tissue culture hood (Thermo Scientific, Waltham, MA). After establishing flow at the desired rate (low rate: 0.60∼0.68 mL/min (1.2–1.36 mm/sec); high rate: 1.6–1.8 mL/min (3.2–3.6 mm/sec) or 2.9–3.0 mL/min (5.8–6.0 mm/sec)) with a CellMax Quad pump (Spectrum Laboratories, Inc.) for about 2 minutes, flow was stopped to allow the growth factor of interest (FGF-2 (Sigma Aldrich, St. Louis, MO), EGF (R&D Systems Inc., Minneapolis, MN) and VEGF (R&D Systems Inc., Minneapolis, MN)) (0.11 mL) to be injected into the inlet. After the injection, the flow was resumed and the flow media collected (two drops/fraction) for the desired time period. The flow pattern was assumed to be sigmoidal based on previous studies [18], [19]. The cartridges were then gently washed with warmed PBS supplemented with 0.3M NaCl (10 mL) followed by one wash with 10 mL PBS and a wash of the whole flow system with PBS (60 mL). The system was returned to the same culture media and flow rates as described under Preparation of BAECs, allowing at least 24 hours before the next experiment. The media fractions collected during the binding experiments were stored at 4°C and analyzed with ELISA kits (R&D Systems Inc., Minneapolis, MN) within the next 24∼48 hours.

### Viscosity measurements

Dynamic viscosity of the test cell culture medium was measured using a DV-II++ Pro Programmable cone-plate viscometer (cone #CPE-40; Brookfield Engineering Laboratories; Boston, MA) according to the manufacturer's instructions. Viscosity measurements were made for a range (375 to 750 sec^−1^) of shear rates (to confirm Newtonian fluid behavior) at room (i.e., 25°C) and physiologic (i.e., 37°C) temperatures.

### Enzymatic treatment

Heparan sulfate expression was measured in static tissue culture dishes and in the flow cartridge by heparinase treatment of cells, collection of the cleaved glycosaminoglycans, and quantitation using a dimethylene blue colorimetric assay [20], [21]. Cells in static culture contained 4.3+/−0.31×10^−6^ µg of heparan sulfate/cell and cells in cartridge hollow fibers contained 1.1+/−0.09×10^−6^ µg of heparan sulfate/cell, reflecting an ∼75% reduction in cell surface heparan sulfate under flow (0.63 mL/min (1.26 mm/sec)).

Heparinase III (0.01 unit/0.11mL, Seikagaku Corp., Japan; 0.2unit/0.11mL, Sigma Aldrich, St. Louis, MO), chondroitinase ABC (0.2 unit/0.11mL, Seikagaku Corp., Japan) and keratanase (0.33unit/0.11mL, Sigma Aldrich, St. Louis, MO) were utilized to observe their effect on growth factor flow and binding. In some experiments, the enzymes (heparinase III, chondroitinase ABC and keratanase) were mixed together as an enzymatic cocktail solution at the above concentrations. Cartridges were treated for 20 minutes at 37°C, washed with warmed PBS (10 mL), and growth factor studies performed as described above.

### Determination of non-specific binding

Non-specific binding of FGF-2 in the system was determined to be primarily due to the inlet reservoir. The reservoir chamber was removed from the cartridge, growth factors were injected into the inlet of the cartridges with a syringe, and flow was initiated. Fractions were collected as they exited the reservoir. Growth factors were measured before injection and compared to the sum of the collected fractions. The difference between the input amount and the amount collected constituted the nonspecific binding in our experiments. For FGF-2 (1.0+/−0.1 ng), the amount retained in the reservoir was 29+/−2.8% of the FGF-2 added (SD, n = 3). Additional nonspecific binding within the hollow fibers was assumed to be minimal.

### Determination of growth factor concentration in outflow

The concentrations of FGF-2, EGF, and VEGF in the collected fractions were measured by ELISA. The flow rate of each experimental run was determined from the total volume collected divided by the total flow time.

### Immunofluorescent staining of BAECs from the bioreactor flow system

To visualize the BAECs cultured in the flow system, cartridges were washed with PBS supplemented with 0.5M NaCl to extrude the endothelial cell lining from the hollow fibers and then the cell linings were fixed with 4% paraformaldehyde (Electron Microscopy Sciences, Hatfield, PA) in PBS for 10 minutes. Three washes with PBS (one minute per wash) followed and the cell linings permeabilized with PBS supplemented with 0.03% Triton and 1% BSA for 3 minutes on a shaker platform at room temperature. The cells were then treated with 10 µg/mL 4′, 6-diamidino-2-phenylindole (DAPI) (Sigma Aldrich, St. Louis, MO) in PBS supplemented with 0.03% Triton and 1% BSA for 20 minutes, followed by three PBS washes for 2 minutes each at room temperature. The cells were then visualized and photographed using a Nikon Eclipse TE 2000E fluorescent microscope (Nikon, Melville, NY) at an excitation wavelength of 350 nm (Figure 1B).

### Model development

The computational model is based on the physical dimensions of the bioreactor although the system is scalable to other desired dimensions. The domain of the simulation is the hollow-fiber portion of the cartridge (Figure 1). The computational model has three coupled parts: (1) the medium flow equations; (2) the convective mass transport equations of growth factor in the flow; (3) the binding kinetics equations on the wall of the fibers [8], [16].

**Figure 2:**
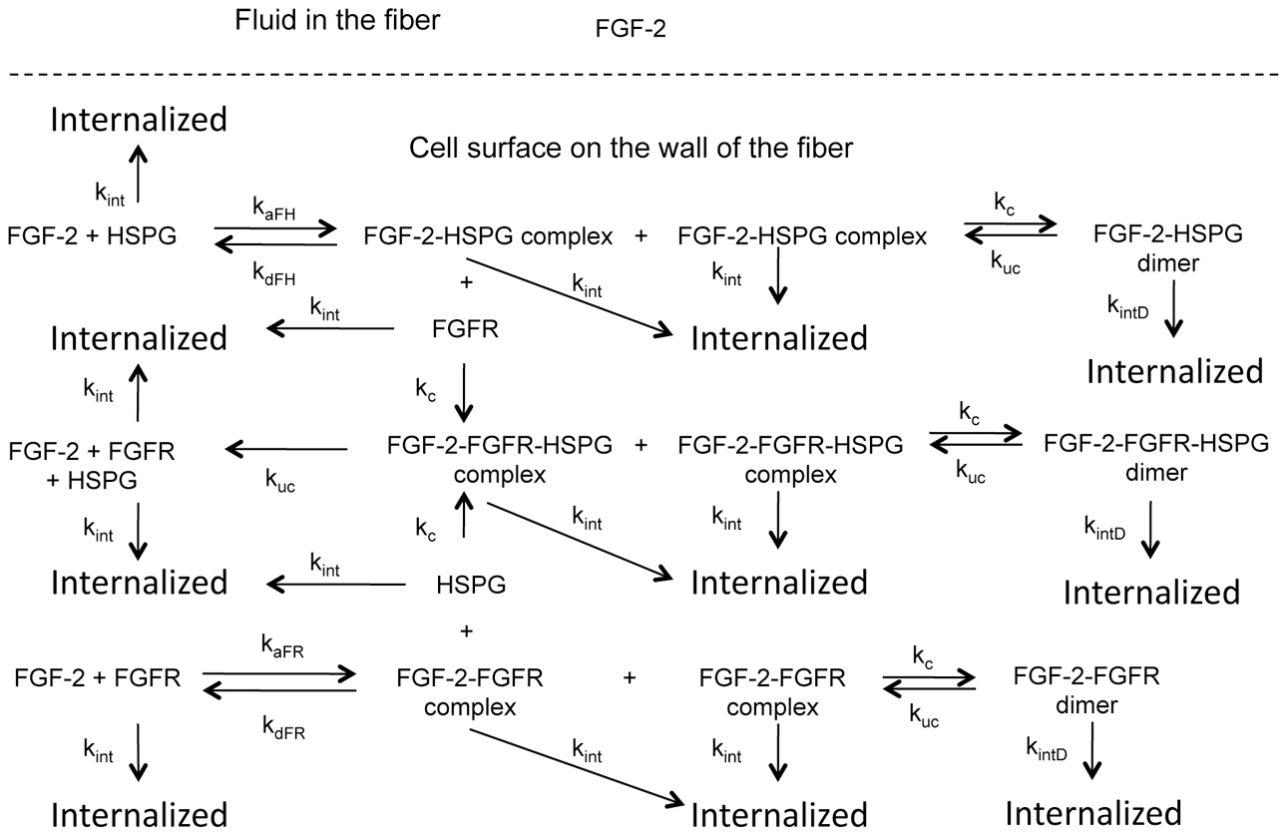
Schematic of reaction pathways on the cell surface. FGF-2 is the only species in the fluid phase with all reactions included in the model occurring on the cell surface and incorporated in the model as boundary conditions.

In order to solve the coupled equations numerically and efficiently, the following assumptions are made: (1) the walls of the hollow fibers are rigid and nonporous; (2) the flow is axisymmetric and laminar; (3) the fluid is incompressible, Newtonian and isothermal; (4) all of the hollow-fiber capillaries within the cartridge have the same dimensions, flow rate, cell densities and entrance conditions; and (5) the cells are packed tightly and distributed evenly on the wall of the hollow-fiber capillaries. Entrance effects of the flow are ignored [22], [23] and, consequently, the flow within the fibers is treated as fully developed flow in which the radial velocity is neglected. A uniform mesh is used. The kinetic pathways are shown in Figure 2 and the equations and parameter values are included in Tables 1 and 2, respectively.

**Table 1:** Equations describing the binding reactions.

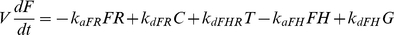	(1)
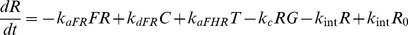	(2)
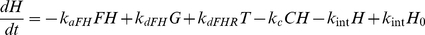	(3)
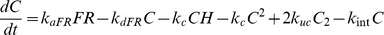	(4)
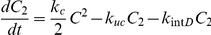	(5)
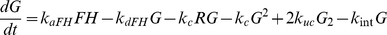	(6)
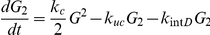	(7)
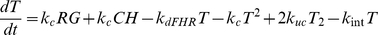	(8)
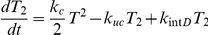	(9)

Cells line the walls of the hollow fiber tube in our model and growth factor can bind to both receptors (R) or HSPG (H) to form complexes (C or G, respectively). These complexes can dimerize (C_2_ or G_2_,) or form heterodimers (T) that can then form higher order complexes (T_2_). The equations that describe the binding reactions are listed as well as the parameters (Table 2) and initial conditions used for the simulations.

The initial condition for the FGF-2 concentration (F) was based on the amount of FGF-2 injected and the volume of inlet reservoir as described in Materials and Methods. The concentration is assumed to be uniform across the entrance. The receptor and HSPG densities were the initial conditions for R and H respectively. All other variables had an initial value of zero.

**Table 2:** Parameter values used in simulations.

Parameter	Value	Parameter	Value
k_aFR_	3.2×10^8^ M^−1^min^−1^ *	k_intD_	0.078 min^−1^ *
k_dFR_	0.28 min^−1^ *	R_0_	1×10^4^ # cell^−1^ &
k_aFH_	1.2×10^8^ M^−1^min^−1^ *	H_0_	2.5×10^5^ # cell^−1^ %
k_dFH_	0.56 min^−1^ &	ρ_cell_	800,000 # fiber^−1^ %
k_c_	0.0024 (#/cell)^−1^ min^−1^ ∧	v	4.7×10^−12^ L cell^−1^
k_uc_	0.6 min^−1^ ∧	ρ_fluid_	1000 kg m^−3^
k_dFHR_	0.018 min^−1^ &	μ	0.00094 Pa·s%
k_int_	0.005 min^−1^ *	D	1.67×10^−10^ m^2^ s^−1^ &

*[47] but scaled to 25°C except for k_int_ and k_intD_.

& [7] but scaled to 25°C except for R_0_.

**∧:** [48].

**%:** measured.

In our experimental system, FGF-2 is injected into the inlet reservoir where it is assumed to quickly reach a uniform concentration. The concentration of FGF-2 in the reservoir is assumed to decrease gradually as fluid is pumped into the reservoir prior to distribution into the capillaries with each pulse cycle as:
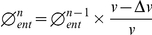
where 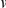 is the volume of the reservoir, 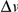 is the volume of fluid flowing into the fibers at each pulse, 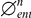 is the current and 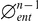 is the previous concentration of FGF-2 in the reservoir. 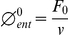, where 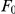 is the amount of FGF-2 injected. The pump pulse cycle was measured experimentally and determined to be ∼36 strokes/min at a flow rate of 1.4 mm/sec.

Pulsatile flow is treated in the following manner. A pulse of fluid volume enters the pre-pump inlet reservoir (0.4 mL volume), from which a continuous flow of fluid having an axial velocity greater than or equal to zero enters the cell-lined fibers in the cartridge. The axial velocity is oscillatory but with only positive terms. Entrance effects are considered negligible [23]. The velocity of the fluid in the axial direction is determined with the following formula [17]:
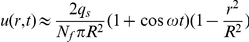
where *q_s_* is the average volumetric flow rate, *N_f_* is the number of fibers inside the cartridge, *R* is the radius of a fiber, ω = 2π/*T* is the angular frequency of the pulsatile flow, and *T* is the pump pulse cycle.

Good agreement between the simulation and experimental results was determined based on two criteria: an amount criterion and a curve-matching criterion. The amount criterion is defined as:
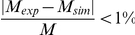
where M_exp_ is the outflow amount of protein determined experimentally, M_sim_ is the outflow amount determined within the simulations and M is the amount of FGF-2 entering the capillary. The curve-matching criterion is calculated in the following way. The FGF-2 exit profile curve is not a continuous curve but is a series of discrete values at different time intervals. This makes use of traditional curve matching algorithms difficult. Our method aligns the initial exit times for the simulations and experiments and then calculates the distance between points on the two outflow curves using the following formula:
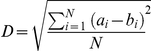
where *N* is the total number of time intervals. 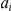 and 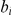 is the amount of FGF-2 exited at the *i*th time interval in experiment and simulation, respectively. The curve-matching criterion is defined as:
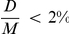
A special program written in C/C++ that operates under Windows XP or Vista operating system has been built for solving this model and has been described previously [16], [17]. The interface allows users to easily set parameters related to the simulation such as FGF-2 injected concentration, flow rate, mesh size, time step, and total simulation time via either configuration text files or from the computer interface. The mass transport of FGF-2 within the fiber is visualized in real time during the simulation process. A Linux version of the software is also available however it lacks a user interface tool and there is no real time visualization. The binary code can be downloaded from www.cs.uky.edu/~czhanb/research.html.

In the simulations there are 800,000 cells/fiber or 16,000,000 cells/cartridge, a value which was obtained from the experimental system. The tolerance for solving the mass transport PDEs was set at 10^−12^. The relative tolerance for solving the kinetic ODEs was set at 10^−8^ and the absolute tolerance was 10^−12^.

### Statistics

All experiments were performed a minimum of three times in independent cartridges. The mean of all replicates ± standard deviation of those replicates is presented except where discrete measurements were used to more closely represent small changes in initial concentration. Significance (p<0.05) was determined using a Student t-test with a two-tail distribution and unequal variance (Excel, Microsoft).

## Results

### Endothelial cells form a uniform and confluent monolayer in cartridge capillaries

Endothelial cells line blood vessels and are the initial entry point for access of blood-borne proteins to the underlying tissue. Our investigations focused on flow and the impact it has on endothelial cell capture of growth factors, which are important regulators of cell and tissue activity. To better approximate the microenvironment of a blood vessel, we seeded bovine aortic endothelial cells into the FiberCell cartridge system and cultured the cells under flow (Figure 1A). Cell viability was confirmed for up to 8 weeks and cell density was ∼0.3×10^6^/cm^2^. The geometry is clearly more similar to *in vivo* than typical cell culture dishes but it was important to obtain a uniform and confluent monolayer of cells within the cartridge system to correctly perform and analyze experiments. To confirm this, cartridges were treated with a high salt wash to extrude the cell-based vessel and the cells were fixed and imaged (Figure 1B). An incision was made at one end to expose the lumen and demonstrate the continuity of the cell layer.

### There is significant capture of FGF-2 under low flow

**Figure 3:**
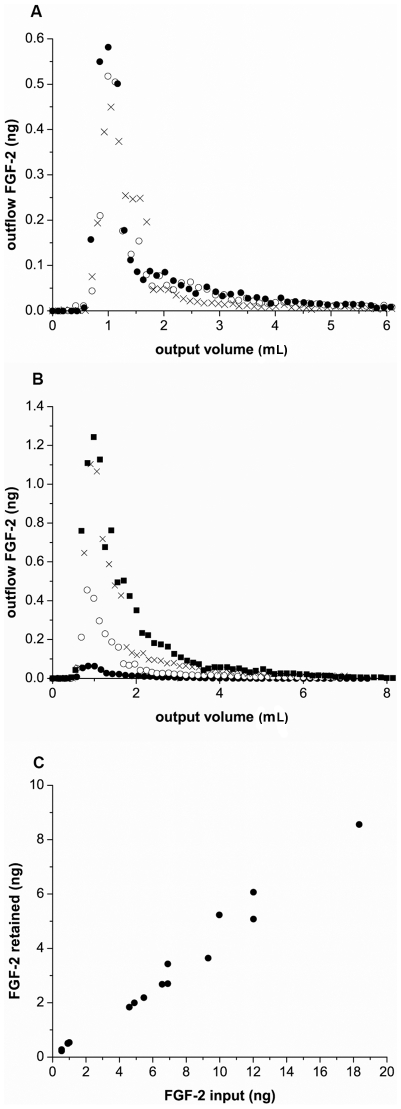
Significant retention of FGF-2 occurs under flow. (A) FGF-2 (5.0+/−0.4 ng) was injected into the inlet reservoir, pumped through the cartridge at 0.65+/−0.01 mL/min (1.3 mm/sec), and measured in the output stream samples from three independent runs on three separate cartridges. The average retention of FGF-2 within the cell-lined cartridge was 40+/−0.5% FGF-2 (mean +/− standard deviation of the three runs shown) with a specific binding of 9+/−2.5% (B) FGF-2 ((•) 0.92 ng , (○) 6.9 ng, (X) 12 ng, and (▪) 18 ng FGF-2) was injected into the initial reservoir, run through the system at 0.64 mL/min, and the FGF-2 in the output stream measured using ELISA. (C) FGF-2 (ng) retained within the cell-lined cartridge versus the FGF-2 (ng) injected into the system is shown. The flow rate for this study varied between 0.60 and 0.67 mL/min (1.2 and 1.34 mm/sec respectively). Results are from individual runs with 9 independent cartridges.

The average fluid velocity in human capillaries is <1 mm/sec [4]. We hypothesized that capture of regulatory growth factors from solution would be significant at these flow rates thereby facilitating growth factor activity. Using the lowest velocity setting with the standard pulsatile pump included with the Cellmax system (∼1.3 mm/sec, ∼0.65 mL/min), FGF-2 (5.0±0.4 ng) was injected into the cartridge inlet reservoir and flow was commenced. As shown in Figure 3, there is a delay in FGF-2 appearance in the outflow corresponding to the time for FGF-2 to travel through the cartridge and exit the system. The majority of FGF-2 added exited the cartridge as a large peak approximately 1 mL (or 1.5 min at this flow rate) after flow was initiated. Non-specific binding within the injection cartridge reservoir was measured directly (31+/−2.5%). Specific binding within the cell-lined hollow fibers accounted for 9+/−2.5% of total FGF-2 added to the cartridge at this concentration and ∼13% of the FGF-2 entering the cell-lined fibers, after taking into account non-specific binding (Figure 3). The results shown in Figure 3A are from three independent experiments conducted using three different cartridges illustrating the reproducibility of the system. Repeat runs conducted using the same cartridge as well as runs using radiolabeled FGF-2 instead of unlabeled FGF-2 both produced similar results (data not shown). The peak appearance time or volume in the outflow from the cartridge was insensitive to FGF-2 injection concentration in the range studied (data not shown). However, the size of the FGF-2 peak correlated with the injection concentration with the highest peak corresponding to the highest concentration of FGF-2 added (Figure 3B).

The accuracy of our measurements took into consideration specific losses that occurred with injection (i.e. tube, syringe, needle, and reservoir). Rather than averaging datasets with variable FGF-2 reservoir values, we therefore present them as discrete results. A plot of total FGF-2 retained at these discrete concentration points shows a dose responsive binding curve, reflecting the linear portion of the binding curve expected at sub-saturation ligand concentrations (Figure 3C).

### Heparinase treatment significantly increases the FGF-2 outflow

Heparan sulfate proteoglycans (HSPG) are ubiquitous molecules found on virtually all cells including endothelial cells and have been shown to regulate heparin-binding growth factor binding and activity in tissue culture [6], [24]–[28]. FGF-2 is a heparin-binding molecule associated with a number of physiologic and pathologic processes [29] and, therefore, the role of HSPG in regulating FGF-2 retention under flow was examined. Although the binding affinity of FGF-2 for HSPG has been shown to be lower than the affinity for the FGF receptor, these HSPG sites can provide up to a thousand fold more binding sites for FGF-2 [6], [24] significantly impacting the cell binding “potential” for heparin-binding growth factors. Cartridges were treated with heparinase, an enzyme specific for heparin and heparan sulfate, and FGF-2 outflow quantified. After heparinase treatment, FGF (∼1 ng) was injected and pumped through the cartridge. Almost 74% of the total FGF-2 added to the system was recovered in the outflow, compared to ∼46% of the total FGF-2 recovered from the non-heparinase treated cartridge prior to subtraction of non-specific binding. The amount of FGF-2 retained in the cartridge after heparinase treatment corresponded to the measured level of non-specific binding and thus indicated no specific binding to cell-lined fibers in the absence of HSPGs (Table 3). In contrast, 25% of the FGF-2 pumped through untreated cartridges was retained after subtraction of non-specific binding. Although FGF-2 can bind to its receptor in the absence of HSPG stabilization, that binding, based on the apparent K_D_ of the receptor for FGF-2 in the absence of heparan sulfate, the lower level of FGFR generally found, and the ligand-receptor exposure time under flow_,_ would be expected to be at least ten-fold lower than in the presence of HSPG [24] and our data certainly support this.

**Table 3:** Heparinase and chondroitinase but not keratanase impact FGF-2 output.

Treatment	FGF-2 input(ng)	% FGF-2 retained	flow rate (mL/min)
control	0.95+/−0.05	25+/−1.7	0.62+/−0.02
heparinase	0.92+/−0.00	0.0+/−2.9*	0.66+/−0.02
chondroitinase	1.73+/−0.68	16+/−4.1*	0.65+/−0.03
keratanase	0.95+/−0.15	20+/−7.5	0.62+/−0.08

Mean +/− standard deviation of at least three experimental runs.

*indicates significantly (p<0.05) different from control.

To ensure that the effect with heparinase under flow was due to the specific removal of heparan sulfate and not a general effect due to enzymatic treatment of the cartridge or the enzyme incubation process, the cartridges were treated with keratanase, an enzyme having no specific known target on these cells. Keratanase, as opposed to heparinase, had no significant effect on FGF-2 retention (Table 3). Interestingly, there was a small but reproducible reduction (∼9%) after chondroitinase treatment on FGF-2 retention compared to control. Chondroitin sulfate proteoglycans are typically found on vascular surfaces but FGF-2 has not been shown to bind directly to chondroitin sulfate [30], [31]. It is not known at this time what the cause for the reduced binding is, although it has been reported that both chondroitin sulfate and dermatan sulfate under certain circumstances are able to influence FGF binding [32]–[34].

### VEGF but not EGF is impacted by heparinase treatment

**Figure 4:**
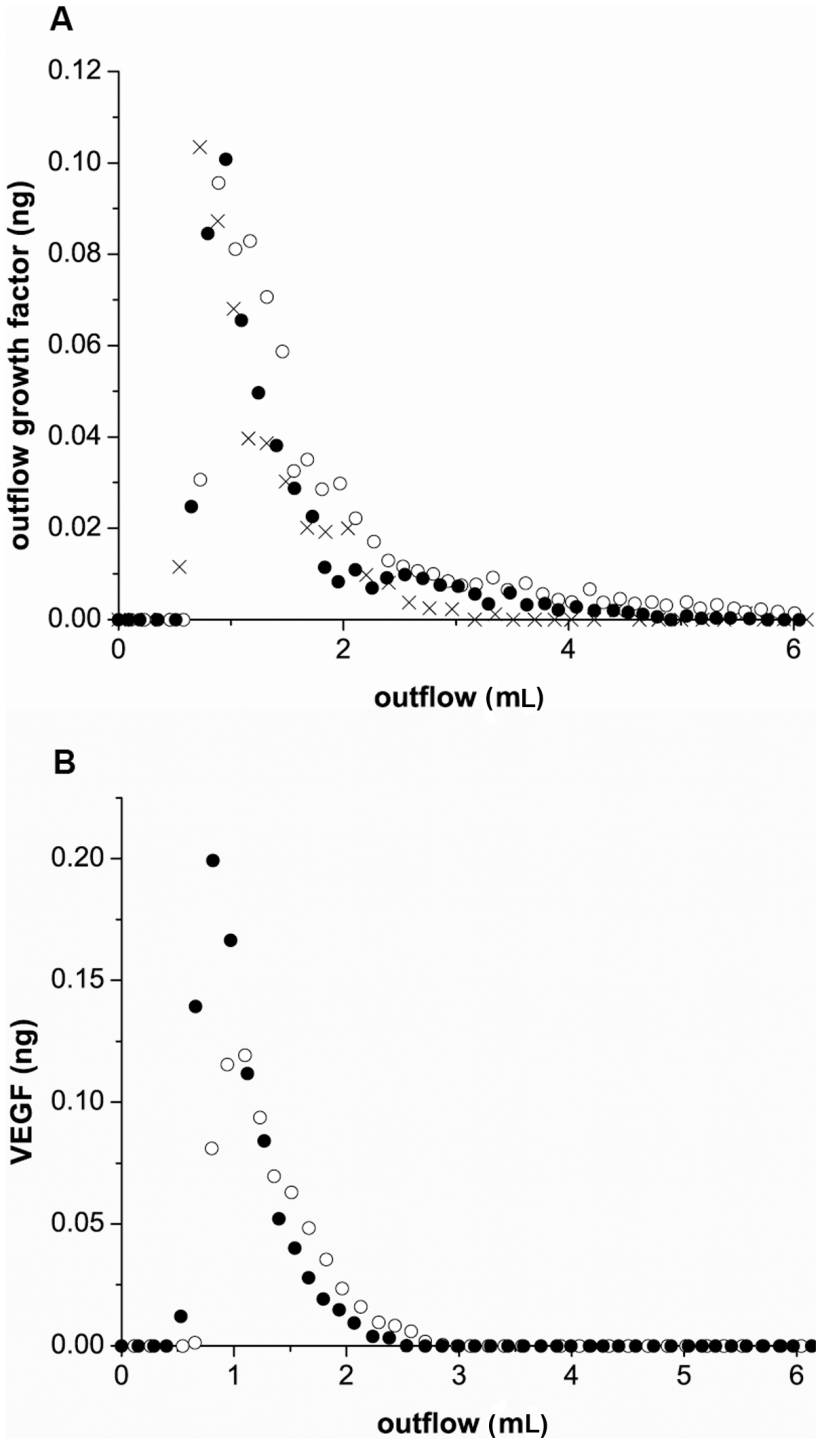
EGF and VEGF are retained under flow. (A) EGF (1.49 ng) was injected into the input reservoir, pumped through the system at 0.61 mL/min (1.22 mm/sec), and EGF quantified in the output flow by ELISA. Data shown are from the same cartridge either untreated (○) or enzyme-treated (•). FGF-2 (1.01ng - x) is shown for comparison. (B) VEGF was injected into the input reservoir of untreated (0.95ng - ○) or heparinase-treated (0.98ng -•) cartridges, run through the system at 0.66 mL/min (1.32 mm/sec), and VEGF quantified in the output flow by ELISA. Data are representative of at least three runs quantified in Table 4.

VEGF, a heparin binding protein, and EGF, which does not bind heparin, were next tested in this system. Both the initial appearance time and outflow volume for the protein as well as the general shape of the outflow peak for both VEGF and EGF were similar to FGF-2 (Figure 4). To ensure that the measured effects seen with heparinase-treatment on FGF-2 retention were due to specific responses of the growth factor to the removal of heparan sulfate and not a general response by all proteins, flow studies were done with VEGF and EGF following enzymatic treatment. EGF retention and outflow were unaffected by treatment with a cocktail of heparinase, chondroitinase, and keratanase (Table 4). Treatment with heparinase without chondroitinase or keratanase also had no effect on EGF retention or outflow (data not shown). In contrast, VEGF showed a significant decrease in specific retention between control and heparinase treated cartridges (16+/−5.8% versus −2.5+/−6.1% VEGF retained) indicating the critical role HSPG can have in heparin-binding growth factor capture under flow. The lack of a change in EGF binding or outflow profile under heparinase treatment is supportive that there are no gross changes in the cell glycocalyx that might impact the shear stress in the system.

**Table 4:** VEGF but not EGF retention is impacted by heparinase (experimental).

Treatment	Growth Factor input(ng)	% Growth Factor Retained	Flow rate (mL/min)
EGF	1.4+/−0.15	19+/−8.1	0.61+/−0.01
+Enzymes	1.6+/−0.17	20+/−7.2	0.62+/−0.01
VEGF	1.2+/−0.19	16+/−5.8	0.66+/−0.00
+Heparinase	1.0+/−0.26	−2.5+/−6.1*	0.65+/−0.02

Mean +/− standard deviation of at least three experimental runs.

*indicates significantly (p<0.05) different than non-enzyme treated case.

### Simulations capture critical properties of process

**Figure 5:**
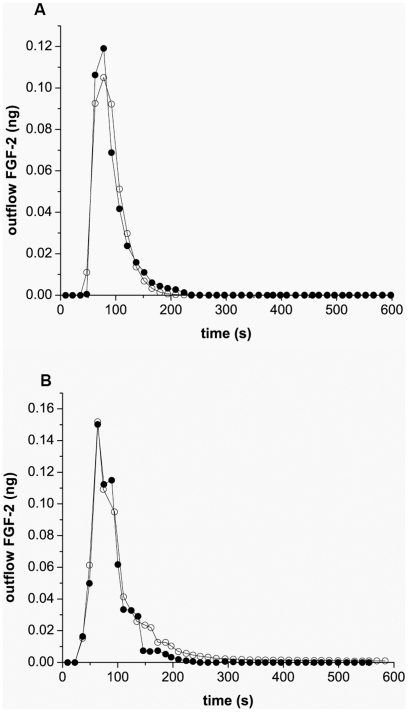
Simulations agree well with FGF-2 outflow measurements. (A) FGF-2 (0.92 ng) was injected into the cartridge reservoir and then flowed through the cell-lined hollow fibers at 0.63 mL/min (1.26 mm/sec), pulsatile flow. FGF-2 collected from the exit fluid (•) is shown. Simulation results based on cells expressing 1×10^4^ FGFR/cell and 2.5×10^5^ HSPG/cell with 32% loss in the entrance reservoir having the same FGF-2 amount injected at the same flow rate (○) are also shown. (B) Similar outflow FGF-2 measurements are shown following FGF-2 (0.92 ng) addition for heparinase-treated (experimental - •) and simulation results with out HSPG (simulations - ○). Simulations were run with cells expressing 1×10^4^ FGFR/cell and 30% loss in the entrance reservoir.

Capture of FGF-2 by endothelial cells within the vasculature is a critical step in growth factor activity and our bioreactor is an excellent tool for investigating the capture process. However, it has limitations with regard to quantification of cellular binding behavior. The cartridges are expensive for short-term experiments and culture time and preparation can be relatively lengthy. Visualization of individual cell behavior within the culture is not feasible. In addition, the ability to predict the capture of molecules by cells under flow has value across a wide range of areas and the development of a flow-based tool for the design and testing of mechanisms related to retention is desireable. Our computer model was designed based on media flow equations and mass transport equations [35] with cell surface reaction equations to reflect the cell-growth factor interactions (see Materials and Methods-Model development). To validate the model, simulations were performed using the variables (ie FGF input concentration and flow rate) specific for an experimental series and a comparison was made. Experimental trials were run in which FGF-2 (0.92 ng) was added to the reservoir, pumped through the cartridge, and outflow collected and analyzed for FGF-2. FGF-2 in the outflow showed a characteristic peak outflow approximately 100s after flow was initiated at 0.63 mL/min (1.26 mm/sec) and 17±6.3% of the input FGF-2 was retained within the cartridge after non-specific binding was subtracted (Figure 5). Simulations performed using the same input FGF-2 value and flow rate were run and comparison was made between the simulations and experimental outflow from control (Figure 5A) or heparinase-treated (Figure 5B) cartridges. We defined good agreement based on two criteria; the amount of FGF-2 recovered and the curve similarity. Criteria one requires the relative difference in FGF-2 outlow from the experimental and simulation studies to be less than 1% while the second criteria compares the actual amounts of FGF-2 exiting from the experimental and the simulation system (see Materials and Methods). We did note that FGF-2 retention with the simulations was very dependent on the level of HSPGs with higher densities resulting in too much retention via HSPG-FGF-2 binding and subsequent FGFR coupling while lower HSPG densities resulted in too little retention (data not shown). Comparison of simulation results with our heparinase-treated data showed fine agreement with regard to our criteria when non-specific loss in the reservoir was subtracted.

### Pulsatile and steady flow results are similar at low flow

**Figure 6:**
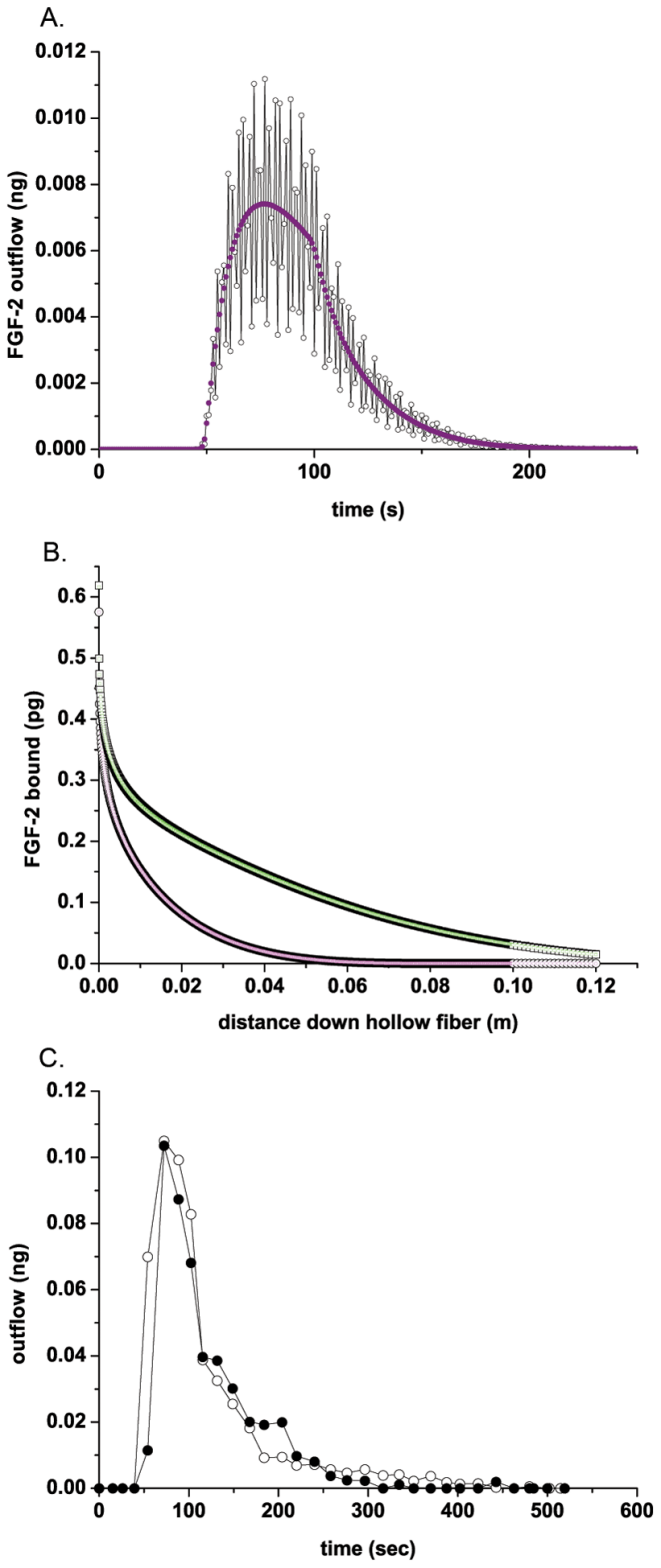
Simulation and experimental comparison between pulsatile and steady flow. (A) Simulation results of FGF-2 in the outflow as a function of time for pulsatile (○) or steady (•) flow, (B) Simulation results of FGF-2 bound along the endothelial-lined hollow fiber as a function of distance at 44 sec (pulsatile (○), steady (pink circle) flow) and at 88 seconds (pulsatile (□), steady (green square) flow) as a function of time, C Experimental comparison of FGF-2 in outflow using pulsatile (○) and steady (•) flow. Simulations and experiments used 1 ng of FGF-2 at a flow rate of 0.6 mL/min (1.2 mm/sec) and pulsatile flow was set at ∼36 strokes/min.

Capillary flow is generally steady, and gradually becomes pulsatile at higher flow rates. We conducted simulations and *in vitro* experiments to compare steady and pulsatile flow at a low flow rate (0.6 mL/min, 1.2 mm/sec) to determine whether our model would predict differences between FGF-2 interactions using steady and pulsatile flow. Simulations predicted no difference in FGF-2 binding at low flow using pulsatile flow conditions versus steady flow in either the FGF binding down the cell-lined hollow fiber (Figure 6A) or in the profile of the outflow (Figure 6B). *In vitro* experiments were performed using a syringe pump for steady flow and the bioreactor's pulsatile flow pump (Figure 6C). FGF-2 outflow measurements indicated no overall change at 0.6 mL/min (1.2 mm/sec) suggesting that, at low rates typical of capillary flow, no significant change in FGF-2 interactions takes place.

### Simulations predict peak FGF-2 binding at entrance to the cell-lined hollow fibers

**Figure 7:**
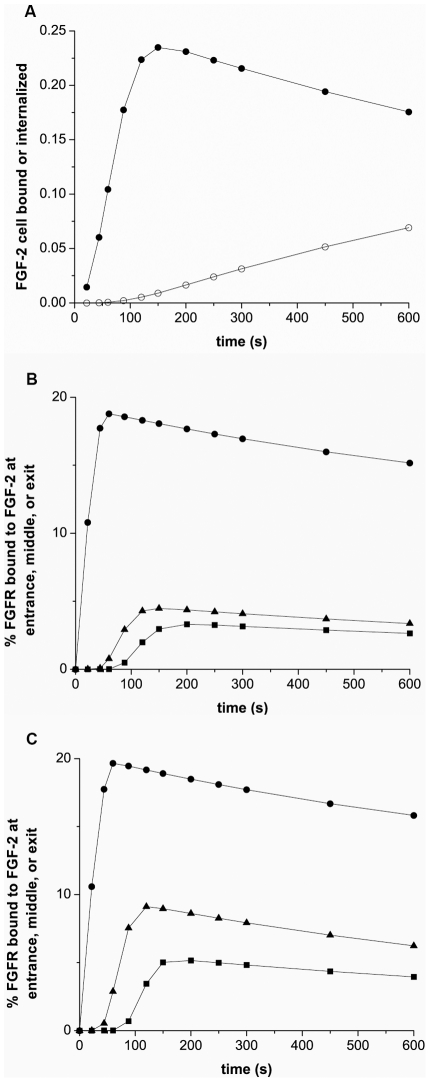
Simulations show FGF-2 binding and internalization under flow. For the simulations, FGF-2 (1 ng) was introduced into the reservoir (30% nonspecific loss) and sent into the cell-lined hollow fibers under pulsatile flow (0.63 mL/min, 1.26 mm/sec). (A) The sum of all cell surface bound FGF-2 (•) and FGF-2 internalized (○) within the cell-lined hollow fiber are shown. (B, C) Plot of % FGFR bound to FGF-2 versus time at the entrance (•), middle (▴) and at the exit (▪) cell when the diffusion coefficient is 1.67×10^−10^ (B) or 1.67×10^−9^ m^2^/s (C). The fluid entering the system is essentially free of FGF-2 by 150s after flow is initiated.

**Figure 8:**
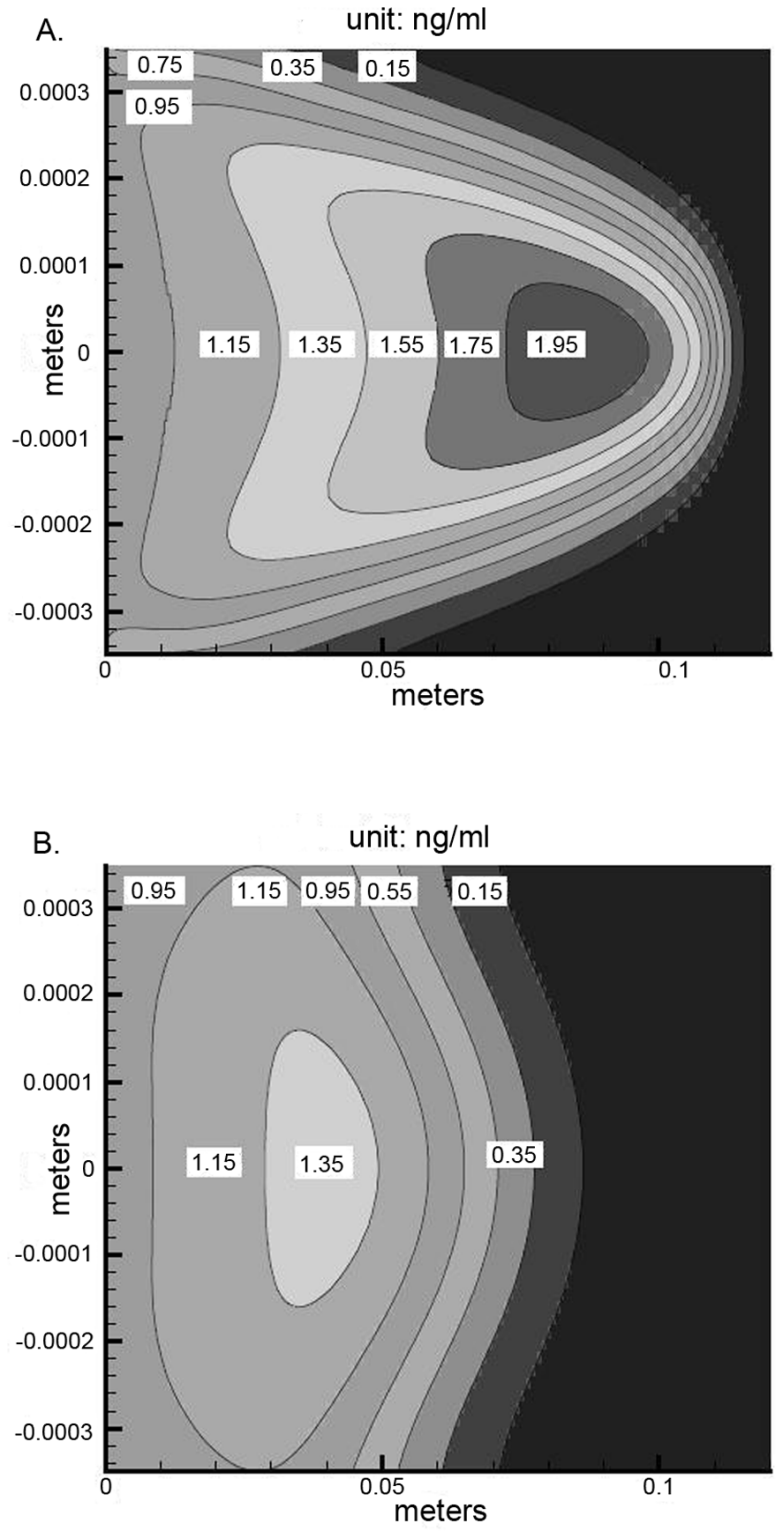
Simulations predict FGF-2 concentration profile in the cell-lined hollow fiber is impacted by diffusion. Grayscale images of FGF-2 concentration within the cell-lined hollow fiber (1×10^4^ FGFR/cell and 2.5×10^5^ HSPG/cell) at 44s after FGF-2 (1 ng) addition from the reservoir (30% nonspecific loss) at 0.63 mL/min (1.26 mm/sec) with FGF-2 having a diffusion coefficient of 1.67×10^−10^ (A) or 1.67×10^−9^ m^2^/s (B). The scale and numbers on the plots indicates the concentration of FGF-2 in ng/mL.

Our experimental system does not allow easy separation between internalized FGF-2 and that bound to the cell surface or visualization of FGF-2 distribution within the cell-lined hollow fiber. Using our computer model we examined how FGF-2 would be distributed with respect to time after flow was initiated (Figure 7). At a relatively low flow rate (0.63 mL/min, 1.26 mm/sec), the FGF-2 in the reservoir had essentially all entered the hollow fibers by 150s and the peak outflow of FGF-2 was evident ∼200s after flow was initiated corresponding to the time when the bulk FGF-2 had exited the hollow fibers. Later times showed cell-bound FGF-2 either internalized or dissociated from the cell surface with little chance to reassociate. The vast majority of binding is predicted to occur near the entrance to the cell-lined hollow fibers as opposed to the middle or end of the fibers (Figure 7B). The impact of time was more pronounced in the front section also as fluid entering the hollow fiber after ∼150s was devoid of FGF-2 (<0.1% of initial FGF-2). Increasing the diffusion rate for FGF-2 in solution by increasing the diffusion coefficient by an order of magnitude is predicted to have a negligible impact on FGF-2 capture in the front of the capillary but increased significantly the FGF-2 bound down the length of the cell-lined hollow fiber. This was due to changes in the depletion zone near the cell-lined walls (Figure 8). After 44s, an FGF-2 depletion zone near the surface was evident which was reduced when the diffusive transport of FGF-2 was increased. The replenishment of FGF-2 near the wall promoted greater FGF-2 binding as complex formation is a second-order process and illustrates the importance of surface depletion in growth factor capture.

### Flow rate impacts FGF-2 binding

**Figure 9:**
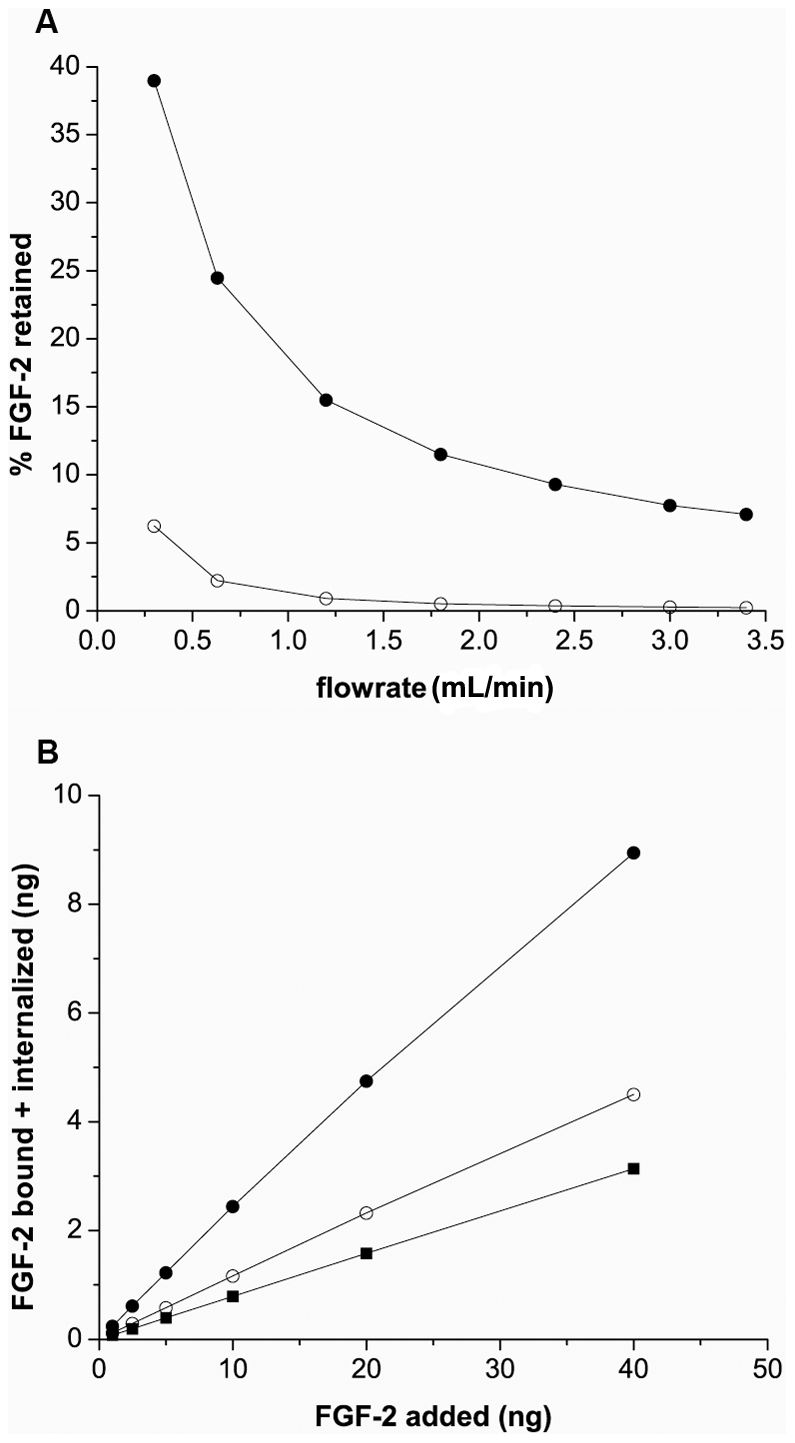
Simulations show reduced binding with increased flow rate. (A) Simulations for control (•), and HSPG-deficient cells (○), were run modeling injection of FGF-2 (1 ng) into the system and run at varied flow rate. 30% non-specific loss of FGF-2 in the reservoir was incorporated. (B) Cell-bound+internalized FGF-2 as a function of injection concentration at 5 min as a function of flow rate is shown. Simulations performed at 0.63 (•), 1.8 (○), and 3.0 (▪) mL/min pulsatile flow(1.26, 3.6, and 6 mm/sec, respectively). Each cell on the cell-lined hollow fiber expressed 1×10^4^ FGFR/cell and 2.5×10^5^ HSPG/cell.

Our simulations indicate that depletion near the cell surface impacts binding and suggests that residence time in the vicinity of the cell surface is important. We therefore looked at how flow impacted cell binding of FGF-2. Simulations predict that cell binding is significantly diminished with increased flow rate (Figure 9A) although the basic result of high binding at the entrance and reduced binding down the cell-lined hollow fiber was consistent across flow rates examined (data not shown). This difference was evident regardless of the concentration of FGF-2 introduced to the system with the difference being more pronounced at higher flow rates (Figure 9B). Reduction in binding due to the loss of HSPG is less evident at higher flow rates where the specific binding was already greatly reduced. This inverse relationship between flow and cell binding is potentially important especially at these relatively low flow rates. The highest rate used in our simulations (∼3 mL/min,∼6 mm/sec) is considerably lower than average arterial flow rates (100–400 mm/sec) in larger vessels of the circulatory system [4] suggesting that, with a short half-life, retention may be relevent only in small vessels with lower velocities. Note that simulations were run to a constant time rather than volume to reduce small fluctutations in retained FGF-2 due to dissociation effects.

Experimentally, we found results that were consistent but not quantitatively exact with this model prediction (Table 5). FGF-2 retention in the hollow fibers was virtually eliminated under medium (∼1.7 mL/min, 3.4 mm/sec) and higher flow rates (3.0 mL/min, 6 mm/sec), a significant reduction compared to binding at 0.62 mL/min (1.24 mm/sec) (Table 3- control group). The simulations, in contrast, did show some level of binding even at the highest level but this likely reflects the idealized conditions used for the model system (i.e. uniform receptor and HPSG densities, free access to coupling between FGF-2 bound molecules). Heparinase treatment showed no significant further reduction in retention at the higher flow rates in agreement with the simulation results.

**Table 5:** Increased flow rate eliminates FGF-2 binding (experimental).

Treatment	FGF-2 input(ng)	% FGF-2 Retained	Flow rate (mL/min)
Control	1.1+/−0.11	6.7+/−4.6	1.7+/−0.10
+Heparinase	1.1+/−0.02	6.7+/−1.2	1.8+/−0.05
Control	0.91+/−0.17	0.5+/−9.1	2.9+/−0.13
+Heparinase	0.95+/−0.25	0.5+/−10	3.0+/−0.03

Mean +/− standard deviation of at least two experimental runs.

Simulations indicated no difference in FGF-2 binding under our pulsatile flow conditions versus steady flow (data not shown). Additional experiments were performed using a syringe pump with steady flow rather than pulsatile flow. FGF-2 outflow measurements indicated no overall change at 0.62 mL/min (1.2 mm/sec) (data not shown). Qualitatively the experimental results agreed with the simulation predictions for the overall effect of flow rate on retention although the model suggested higher retention levels for the control case and closer agreement between control and heparinase at both higher flow rates.

### Changes in FGF-2 affinity for HSPG are predicted to have a larger impact on retention than similar changes in affinity for FGFR at physiological cell densities

**Figure 10:**
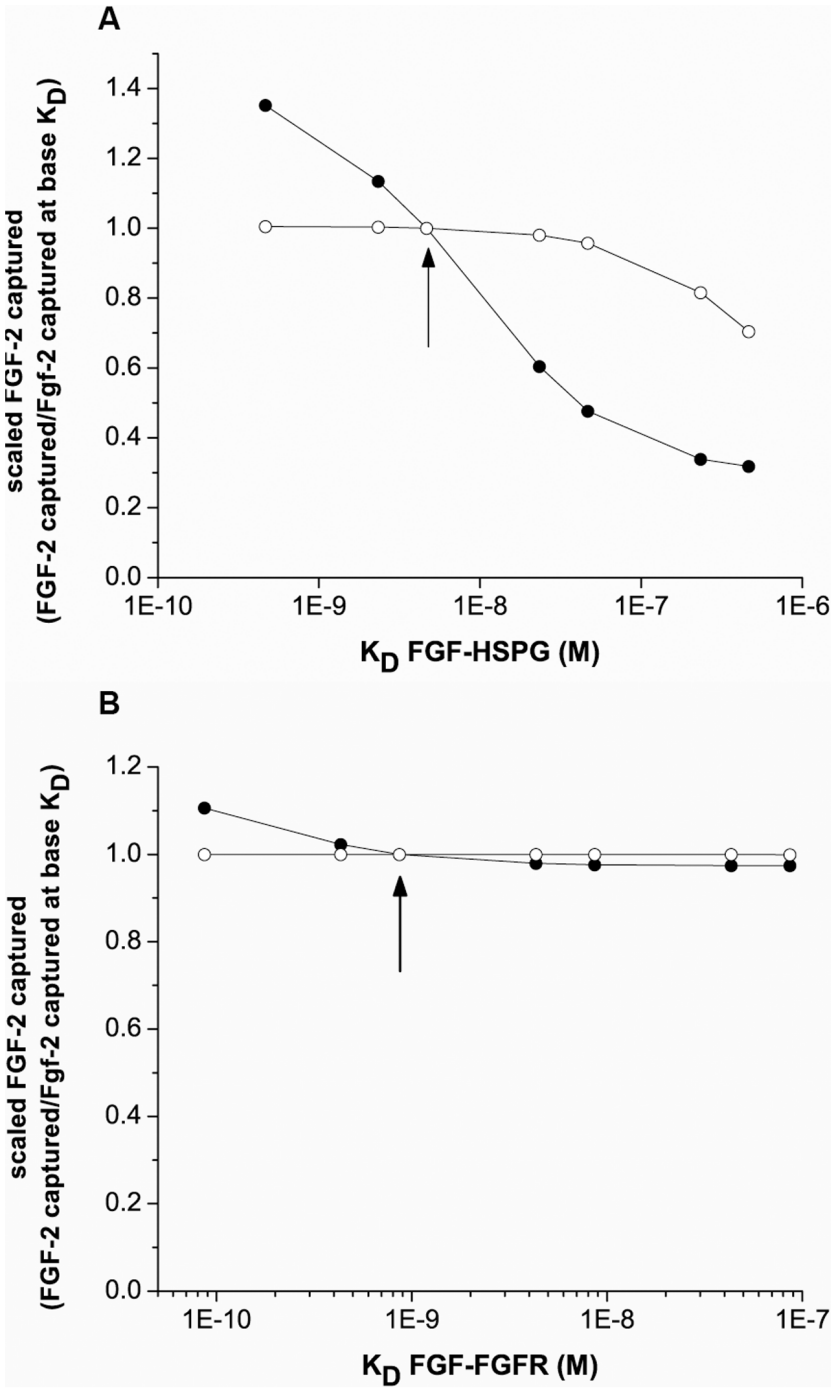
Simulations predict binding affinity of FGF-2 for HSPG impacts FGF-2 capture more than affinity for FGFR. (A) The affinity of FGF-2 for HSPG was varied in simulations by changing the association rate constant (•) or the dissociation rate constant (○). (B) The affinity of FGF-2 for FGFR was varied by changing the association rate constant (•) or the dissociation rate constant (○). The FGF-2 captured within the cell-lined hollow fiber (bound or internalized) at the given *K*_D_ value after 5 min. was scaled by that same value from simulations using the base case *K*_D_ value (Table 2). Arrow indicates base case *K*_D_.

FGF-2 binding affinity and concentration, along with binding partner density, regulates the capture process for FGF-2 from the fluid phase. We therefore examined using our simulations how varying the affinity of FGF-2 for either HSPG (Figure 10A) or FGFR (Figure 10B) while holding all other parameters at their baseline value would impact retention. Decreasing the affinity (i.e. increasing K_D_) for HSPG had a dramatic effect on retention reducing it to 40% of baseline capture at the lowest value examined. The association rate constant had a greater impact than the dissociation rate constant although both followed similar trends. Somewhat surprisingly, increasing the affinity of the interaction by reducing the value of the dissociation rate constant of FGF-2 for HSPG did not alter FGF-2 binding likely due to the strong coupling present between FGFR and HSPG in the presence of FGF-2, making strict HSPG-dissociation somewhat irrelevant. For the same reason, FGF affinity for FGFR did not have a strong impact on FGF-2 capture since the vast majority of FGF-2 interacting with FGFR was via FGF-2-HSPG coupling.

### Simulations predict binding site density is critical for FGF-2 retention

**Figure 11:**
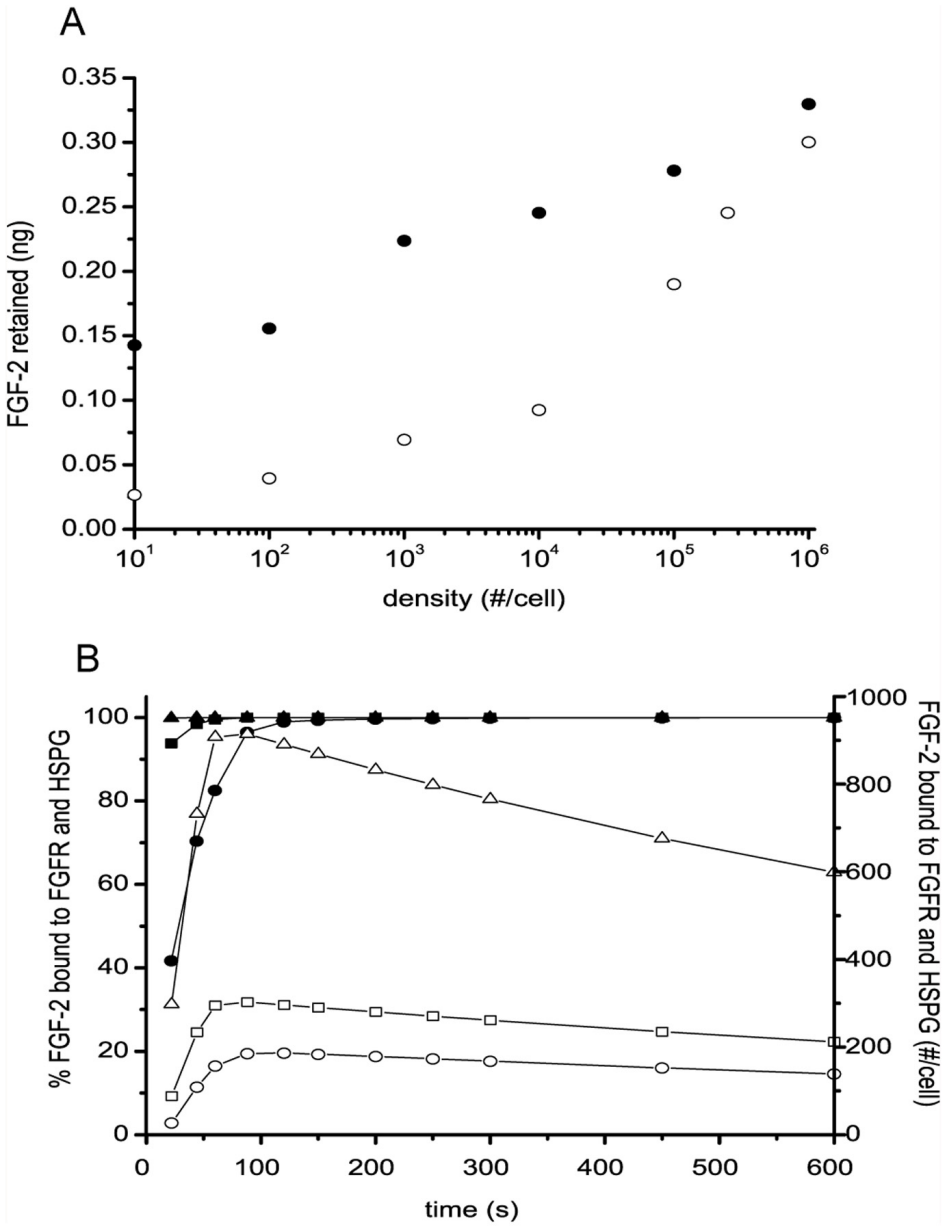
Simulations predict cell surface density impacts FGF-2 retention. Simulations were run for FGF-2 (1ng) added to the system (30% non-specific loss) at 0.63 mL/min pulsatile flow (1.26 mm/sec) for 5 min. (A) Cells expressed either 1×10^4^ FGFR/cell and variable densities of HSPG (○) or 2.5×10^5^ HSPG/cell and variable densities of FGFR (•) on the cell-lined hollow fibers. The amount retained within the system (bound, internalized, and fluid phase FGF-2) is shown. (B) Cells expressed 1×10^4^ FGFR/cell and 2×10^3^ (•,○), 2×10^4^ (▪,□), or 2×10^5^ (▴,▵) HSPG/cell on the cell-lined hollow fibers and simulation results correspond to entrance cell value at a given time. Filled symbols correspond to % of FGF-2 bound to FGFR which are simultaneously bound to HSPG and open symbols correspond to the #/cell of FGF-2 bound to FGFR and HSPG.

Cells typically express significantly more HSPG than FGFR and we next asked how varying the cell surface densities of these binding sites would impact FGF-2 capture. In the absence of FGFR, a typical density of HSPG in our cartridge (2.5×10^5^ #/cell) resulted in significant binding of FGF-2 in the absence of FGFR that is essentially doubled when FGFR density is 1×10^6^ #/cell, a two-fold increase in binding sites (Figure 11A). FGFR typically are expressed at densities of approximately 1×10^4^ #/cell thereby keeping the primary signaling receptor at a controlled level. This is predicted to result in an order of magnitude less overall FGF-2 binding than that found at typical HSPG levels but which is increased in a similar way when HSPG are present. The combination of the two surface binding sites (FGFR and HSPG) is critical. For example, when 1.0×10^4^ FGFR are present, the retained FGF-2 is increased to ∼0.25ng from a value of ∼0.14ng without the FGFR. Looking at cell binding at the entrance of the cell-lined hollow fiber as a function of time after FGF-2 has been introduced with constant FGFR (1×10^4^ #/cell) and variable HSPG, we found that there was a significant increase in bound FGF-2 at the higher HSPG (1×10^5^ #/cell) when compared to the lower values and that the FGFR binding was essentially all coupled to HSPG (Figure 11B). When there are fewer HSPG, there is a lower percentage of coupled binding at least at earlier times as well as lower overall FGFR complexes.

### Simulations predict coupling is key to effective capture of FGF-2

**Figure 12:**
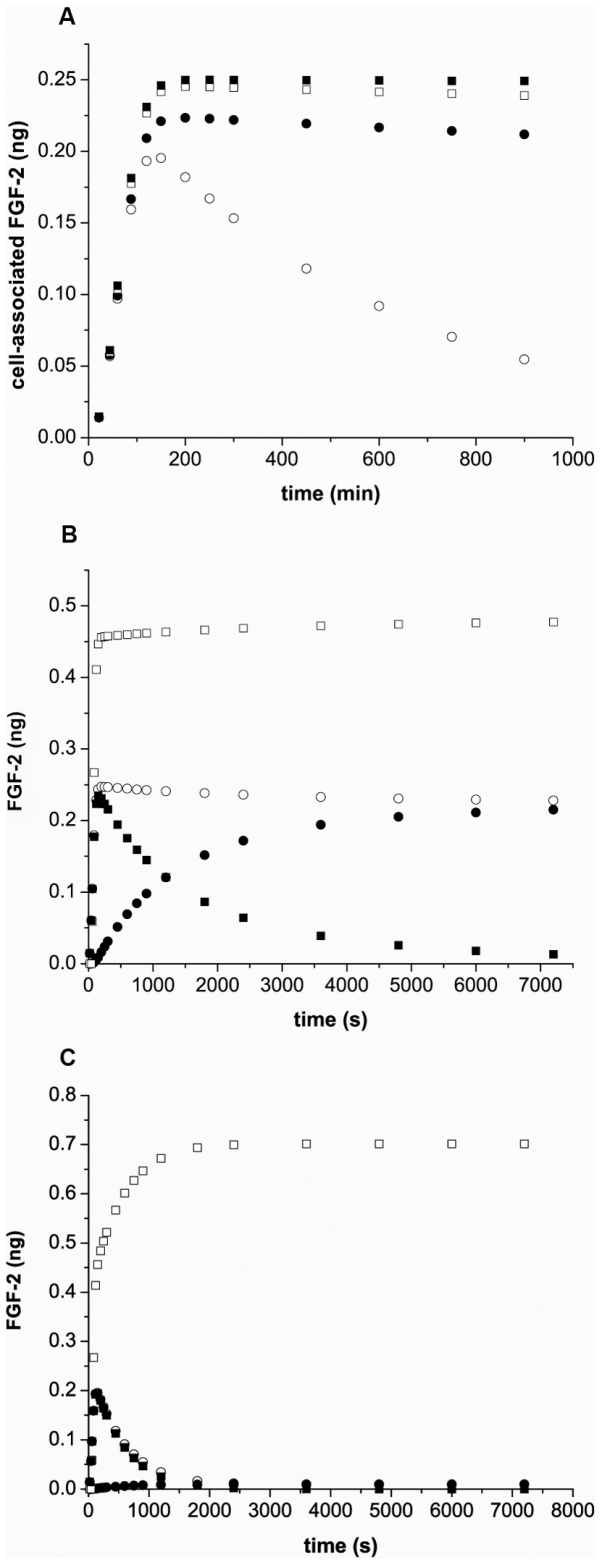
Simulations indicate coupling is critical for FGF-2 retention. (A) FGF-2 bound on cell surfaces plus internalized FGF-2 as a function of time for k_c_ values of 0 (○), 0.0001(•), 0.001(□), and 0.1(▪) (#/cell)^−1^ min^−1^; (B,C) FGF-2 bound (▪), internalized (•), bound plus internalized (○) and exited (□) under flow with k_c_ = 0.0024 (B) or 0 (C) (#/cell)^−1^min^−1^ following addition of FGF-2 (1ng) at 0.63 mL/min (1.26 mm/sec) pulsatile flow(30% non-specific loss). Capillaries were simulated to include 1×10^4^ FGFR/cell and 2.5×10^5^ HSPG/cell on the cell-lined hollow fibers. 300s corresponds to the time when essentially all of the FGF-2 has entered the hollow fiber from the reservoir.

The results with the FGF-2-HSPG affinity simulations and the density studies indicated the importance of coupling in facilitating effective FGF-2-FGFR interactions. We next looked at how varying the coupling rate constant impacted binding and internalization using simulations (Figure 12). In the absence of HSPG-FGFR coupling (k_c_ = 0), there is a reduction in peak binding of FGF-2 and the majority of FGF-2 bound is not internalized but dissociates and exits from the system in the outflow. Even with a low level of coupling, the FGF-2 binding and internalization is dramatically increased until a peak effect is seen with k_c_ = 0. 01 (#/cell)^−1^ min^−1^. If we looked at later times in the simulation (Figure 12B), we would find that a large fraction of the FGF-2 injected is bound during the initial pass and that this bound FGF-2 is largely internalized with little exiting the system. If coupling between HSPG and FGFR is eliminated (Figure 12C), this is not the case. In this scenario, the cells bind a smaller but still significant level of FGF-2 during the initial pass but this FGF-2 is not retained and nearly all of the FGF-2 captured ultimately exits the system in the outflow.

**Figure 13:**
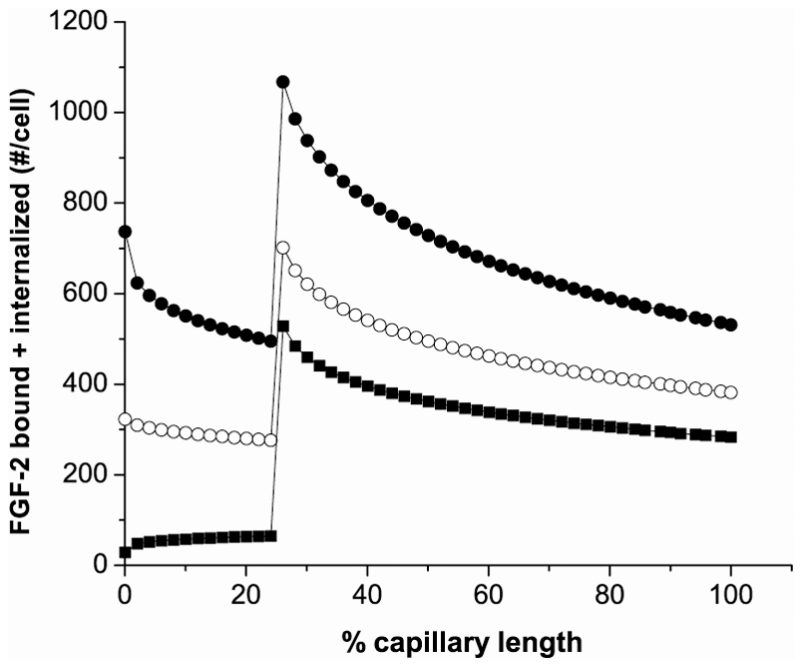
Simulations predict both FGFR and HSPG contribute to retention through FGF-2-mediated coupling. In these simulations, HSPG (2.5×10^5^ #/cell) were expressed on the cell-lined fibers along the entire chamber while FGFR (1×10^4^ #/cell) were expressed only in the cells found in the final 75% of the hollow fiber. FGF-2 (1ng) was added at time 0 (30% loss in the reservoir) at 0.65 (•), 1.3 (○), and 2.6 (▪) mL/min pulsatile flow (1.3, 2.6, and 5.2 mm/sec respectively). Cell-bound+internalized FGF-2 after 5 min of simulation time is shown.

To further illustrate the importance of the coupling process, simulations were performed with cell-lined hollow fibers having only HSPG (2.5×10^5^ #/cell) in the front 25% of the tube and both FGFR (1×10^4^ #/cell) and HSPG (2.5×10^5^ #/cell) in the back 75% of the fiber (Figure 13). The entrance area (front 25%) did not include internalization of FGF-2 by HSPG modeling an ECM-like section, however, the overall outcomes are not significantly changed when internalization is included (data not shown). HSPGs in this front section were able to capture FGF-2 but there is a significant rise in retention in the back section where both HSPG and FGFR are present. This is not simply due to the increase in binding sites due to the addition of FGFR as increasing HSPG by an equivalent level to that of the HSPG plus FGFR did not lead to the same increase in retention (data not shown). Moreover, this increase in retention is lost when the dissociation rate for FGF-2-FGFR-HSPG is reduced to that of FGF-2-HSPG and only nominally increased when the coupling rate is eliminated, reflecting the increased affinity of FGFR compared to HSPG for FGF-2 (data not shown). The effect is evident at both low and high flow rates.

Finally, we used simulations to ask whether dissociation from HSPG in an ECM-like section could lead to increased binding downstream due to slow dissociation of the growth factor and prolonged availability of the growth factor for downstream binding. When the HSPG density in the front 25% zone was increased to 5×10^6^ HSPG/cell, a large increase in overall retention of FGF-2 in the front section was evident resulting in a decrease in FGF binding in the HSPG-FGFR section (back 75%) due to a depletion of FGF-2 in the fluid zone near the cells. This was evident at both 5 (Table 6) and 10 min (data not shown). In contrast, a low level of HSPG (5×10^4^ or less) in the entrance section did not lead to significant binding in this zone and results in increased binding of FGF-2 in the final 75% section. FGF-2 in the fluid phase was at a higher concentration at later times after FGF-2 injection when there were more HSPG in the front section due to dissociation from the HSPGs; however, under flow conditions, this dissociated FGF-2 is not predicted to grow to a high enough concentration to meaningfully impact downstream receptor binding. This is an important difference between flow and static culture studies.

**Table 6:** Simulations predict effect of entrance HSPG zone on FGF-2 capture at 5 min.

	HSPG Density in Front 25% of Cell-lined Hollow Fiber		
	5×10^6^	5×10^5^	5×10^4^
Total FGF-2 Retained (ng)	0.39	0.34	0.31
FGF-2 Bound (ng) (Front 25%)	0.16	0.063	0.0022
FGF-2 Bound (ng) (Back 75%)	0.17	0.24	0.28
FGF-2 Internalized (ng) (Back 75%)	0.013	0.017	0.022
FGF-2 in Fluid Phase (ng)	0.029	0.022	0.008

## Discussion

Circulation is an obligatory process for the maintenance of human life. The proper balance of solid and fluid components, flow and pressure, and chemical content are all tightly regulated to maintain homeostasis. Within these limits, however, wide fluctuations can occur. The effects of the regulatory processes that are in place to deal with these fluctuations are not well characterized. Often the overall effects can be easily measured but not the changes in the microenvironment that come together to drive these effects. Although traditional tissue culture studies have added a wealth of knowledge in such areas, they often lack the capability to emulate the *in vivo* environment. In the study of the effect of flow in regulating vessel wall interactions, for example, three-dimensional studies can provide valuable information. Three-dimensional studies have been used previously to measure the effects of flow on cell populations [18], [36]–[39]. We have chosen such an approach to measure the effect of flow on heparin binding protein delivery. By employing a single pass method to focus on the initial growth factor-vessel wall interaction we were able to more directly measure the effect of flow on the bioavailability of these growth factors. We measured substantial binding of all growth factors (FGF-2, VEGF, and EGF) at the lowest flow rate tested (0.61–0.66 mL/min, 1.22– 1.32 mm/sec). Had a traditional two-dimensional approach been used instead, these factors would have had few limitations on their rebinding potential since in a closed system they would not be subject to the flow that would remove them from the vessel as is typical of normal circulation. In the case of the heparin binding proteins (FGF-2 and VEGF), removal of heparan sulfate sites via enzyme digestion resulted in a significant increase in growth factor outflow (i.e. non-retention within the vessel), suggesting an important regulatory role for these proteoglycans in ligand capture. This is not necessarily surprising given the large number of binding sites these proteoglycans provide on normal cell surfaces. Certainly, it has been shown by us and others that HSPGs are important regulators of FGF-2 binding to FGF receptors in tissue culture [28], although not essential for the interaction [6], [24], [27]. Their importance with regard to capture under flow has, however, not been shown previously and suggests a critical role in the circulation.

An equally significant influence on FGF-2, VEGF, or EGF binding, regardless of heparin binding characteristics however, was the flow rate. By increasing the flow rate by less than a factor of three (∼1.8 mL/min, 3.6 mm/sec) a significant increase was seen in growth factor outflow, reflecting the absence of specific binding taking place on vessel surfaces. A higher flow rate (∼3.0 mL/min, 6 mm/sec) showed no further increase in FGF-2 outflow above that observed at the medium flow rate with both showing retention levels equivalent to that evident in the absence of heparan sulfate. This correlation of flow rate and outflow of growth factors suggests a strong regulatory effect and an environment in the bloodstream that reduces the probability of capture significantly at flow rates typically measured in arteries [4]. Although pulsatile flow is undoubtedly important in increasingly larger vessels and higher flow rates, both simulations and experiments showed that at the low flow rate typical of capillaries it had no significant effect on FGF-2 interactions when compared to steady flow.

The removal of chondroitin sulfate created a small but significant increase in FGF-2 outflow. This is interesting since a number of published findings found no significant affinity between FGF-2 and chondroitin sulfate [30], [31]. It is possible that under flow conditions subtle changes in chondroitin sulfate modifications allow for some weak interaction. Others have reported the ability of FGF-2 to bind chondroitin sulfate under certain circumstances [32]–[34]. EGF binding was, however, unaffected by treatment with a heparinase, chondroitinase and keratanase cocktail suggesting the chondroitinase effect was not universal. How this effect is manifest is currently under further study.

The minimum size of capillaries has been shown to be relatively fixed across species regardless of size [40] and is a basic assumption in the general model of allometric scaling laws proposed by West *et al* [41]. This suggests an optimum environment for the exchange of gases, nutrients, and the removal of waste products that is likely rooted in fundamental physical laws. In order to best make use of these environmental conditions blood flow must also be optimal. Our data demonstrate an inverse correlation between flow rate and probability of capture. Although the presence of heparan sulfate is crucial to FGF-2 capture at low flow rates, at higher flow rates the overriding regulator seems to be the flow rate itself which, based on our results, would all but preclude efficient FGF-2 binding to vessel walls in a single pass under all but the slowest flow conditions. The expectation of lower binding at increasingly higher flow rates might be somewhat expected but the relatively small increase in flow rate required to ablate binding was surprising.

Other influences, such as viscosity, and the presence of competing molecules were not addressed in this work. These are ongoing studies as we begin to add complexity to the system so as to form even more accurate models of circulation. The advantage of this method is that the conditions can be monitored and controlled much as two dimensional culture systems can be but include the three dimensional architecture and flow characteristics that are part of normal blood flow. This approach has obvious potential in the testing of both endogenous molecules and pharmaceuticals in order to provide a better perspective of molecular interactions in the microenvironment of blood vessels.

The importance of HSPGs in FGF-2 binding and signaling has been shown in many systems [6]–[11] and is a generally accepted feature for heparin-binding growth factors. Our work builds upon those studies and shows the critical importance of HSPGs in FGF-2 capture under flow (Figure 3). In this paper, we explore the impact of this critical component in detail using our computational model and show the parameters that regulate this process. In particular we show that the two-step coupling process and the accompanying decrease in dissociation are essential for effective retention of FGF-2 in a flow situation.

HSPG can mediate both the heparin-binding growth factor-receptor interaction at the cell surface and the accumulation and storage of these growth factors in the extracellular matrix [42], [43]. Removal of HSPG from the cell surface by enzymatic digestion greatly impairs FGF-2 activity *in vitro* and inhibits neo-vascularization *in vivo* [27], [28], [44]. HSPG interacts with FGFR directly [45], [46] and FGF-2 binding to cell surface HSPG can facilitate FGF-2 binding to FGFR, which in turn can result in activation of intracellular signaling cascades. Using our simple model under flow, we show in several ways that the coupling step is critical for FGF-2 retention. Elimination of coupling or decreasing the rate constant describing that interaction has a dramatic effect on both FGF-2 bound and internalized with essentially no internalization or effective binding when coupling is eliminated (Figure 12). Reducing the density of HSPG (Figure 11) or the affinity of FGF-2 for HSPG (Figure 9) significantly reduces the amount of FGF-2 bound to both the cell surface and to FGFR. In addition, simulations with only low levels of HSPG (Figures 11, 12 – entrance zone) or FGFR (data not shown) do not exhibit high retention but, when both HSPG and FGFR are present (Figure 13), the combination of both increases retention. This is evident independent of flow rate. The ability of flow to regulate the level of binding suggests how crucial the presence of HSPG is on the vessel wall, in order to increase the probability of capture of heparin-binding molecules especially given the short half-lives of some growth factors in circulation.

Under the flow condition, simulations predict that the majority of FGF-2 binding occurs at the entrance to the cell-lined hollow fiber (Figure 7). In our simulations set up to match the experimental conditions, FGF-2 enters at its highest concentration and thus is most likely to bind under those conditions. Once binding occurs, there is a depletion of FGF-2 in the fluid phase near the cell surface (Figure 8). Under flow, this zone can be replenished via diffusion as increasing the diffusion coefficient increases the concentration in this zone (Figure 8) and ultimately leads to higher binding down the cell-lined hollow fiber. We had postulated that FGF-2 bound in the entrance zone of the cell-lined hollow fiber would eventually dissociate and rebind further down the tube but this does not appear to be the case. Even when binding is extremely high at the entrance, FGF-2 that dissociated from the entrance was not in high enough concentration to impact downstream binding and was eventually washed out of the system (data not shown). In a non-flow system this would likely not be the case and exemplifies the importance of including flow in studies.

In conclusion, a simulation program previously developed by us but enhanced for our specific cell investigations of FGF-2 binding under flow [16], [17] performed well when compared to our experimental endothelial cell-lined bioreactor. Our simulations suggest that: (1) The amount of FGF-2 bound to FGFR is dominated by HSPG and the coupling rate constant, and this triad (FGFR-HSPG-FGF-2) is the key to FGF-2 capture; (2) The amount of FGF-2 bound is proportional to the diffusivity of the growth factor in solution and inversely proportional to the flow rate; (3) Flow rate and diffusivity will affect the FGF-2 outflow profile and the distribution of FGF-2 bound along the cell-lined hollow fiber wall; (4) The majority of FGF-2 binding occurs in the entrance zone of the cell-lined hollow fiber; and (5) most FGF-2 effectively bound by FGFR and HSPG will be internalized rather than dissociated. The simulation environment can provide additional information and insight into capture of FGF-2 that is not easily accessible from experimental work. We have applied the model to our *in vitro* bioreactor system but it has potential to be used for other growth factors as well as other cell systems where flow and capture are pivotal such as in drug and biologicals delivery testing.
